# Treatment of Community-Acquired Pneumonia: Are All Countries Treating Children in the Same Way? A Literature Review

**DOI:** 10.1155/2017/4239268

**Published:** 2017-11-06

**Authors:** Daniele Donà, Dora Luise, Liviana Da Dalt, Carlo Giaquinto

**Affiliations:** ^1^Pediatric Infectious Diseases Division, Department for Woman and Child Health, University of Padua, Padua, Italy; ^2^Pediatric Emergency Department, Department for Woman and Child Health, University of Padua, Padua, Italy

## Abstract

**Background:**

Pneumonia represents an important threat to children's health in both developed and developing countries. In the last 10 years, many national and international guidelines on the treatment of pediatric CAP have been published, in order to optimize the prescription of antibiotics and limit their cost and side effects. However, the practical implementation of these guidelines is still limited.

**Main Text:**

We analyzed the current recommendations for the therapy of pediatric community-acquired pneumonia (CAP) that all converge on the identification of aminopenicillins and beta-lactams as the optimal treatment for CAP. We also conducted a review of the current literature on antibiotic regimens used for pediatric CAP to identify the current state of guidelines implementation in different settings. We selected 37 studies published from 2010 to 2016, including both retrospective and prospective studies, mainly cross-sectional and hospital based. The results show a global heterogeneity in the antibiotics prescription for pediatric CAP, with application of guidelines varying from 0% to more than 91% and with important differences even within the same country.

**Conclusions:**

Our review has demonstrated that the implementation of the guidelines is still limited but also that achieving the optimal prescription is possible and can be done in both developed and developing countries.

## 1. Introduction

Pneumonia is the single greatest cause of death in children worldwide, with an estimated 1.3 million deaths in 2011 and more than 90% occurring in developing countries [1–3]. It is responsible for 4% of deaths in newborns and 14% of deaths in pediatric patients [4]. The incidence of CAP is lower in developed countries: in the US it is about 35–40/1000/person-years in children < 5 years old, 20/1000 person-years in children 5–10 years old, and 10/1000 person-years in children > 10 years old. Despite this, approximately 50% of children with CAP < 5 years old, 20% between 5–10 years old, and 10% of children > 10 years old need to be hospitalized [5]. These numbers demonstrate the burden that CAP represents for society and for economic healthcare resources.

## 2. Materials and Methods

In the first part of the study, we compared the latest national and international guidelines on pediatric CAP, including all those who were published since 2005 to 2016, focusing on their recommendations for first-line therapies.

Then we performed a search on PubMed and Scopus databases, looking for studies published from 2010 to 2016 about CAP antimicrobial therapy in children, trying to get data from as many different countries as possible. We also performed hand-search of references of relevant articles. Our search included both retrospective and prospective studies, mainly cross-sectional and hospital based, including both inpatients and outpatients. All of them except for one [6] included pediatric patients only.

To get a more extensive review of CAP prescribing behavior, for those countries where specific studies on antimicrobial prescriptions for CAP were not available, a search for articles on antimicrobial prescriptions in pediatric age groups was performed. All articles including CAP as reason for treatment were included.

## 3. Results and Discussion

### 3.1. Different Countries, Same Pathogens

Organisms responsible for CAP vary stratifying children by age because of the developing immune system and age-related exposures: viruses or mixed infections are more common amongst younger patients (children under 5 years of age), while exclusive bacterial origin and atypical etiology (mainly* Mycoplasma pneumoniae* and* Chlamydophila pneumoniae*) are more often identified in older children [7, 8].* S. pneumoniae* and* Haemophilus influenzae* are the commonest bacterial pathogens isolated in children under five years with CAP accounting for 30%–50% and 10%–30%, respectively [9]. Around 50% of deaths due to pneumonia are attributable to these organisms [10].

Viral etiology has been documented in up to 80% of CAP cases in children younger than 2 years and much less in older children (10–16 years). The most frequently identified viral pathogen in younger children is* Respiratory Syncytial Virus* (RSV), rarely detected in older children. Less frequent are* Adenoviruses*,* Bocavirus*,* Human Metapneumovirus*,* Influenza A* and* B Viruses*,* Parainfluenza Viruses*,* Coronaviruses,* and* Rhinovirus*. Up to 33% of hospitalized children are simultaneously infected by 2 or more viruses. Mixed infections (both of viral and bacterial etiology) have been documented in 2–50% of children with CAP, more frequently in inpatients, which are more seriously ill than outpatients [3, 11].

Atypical pneumonia caused by different pathogens is characterized by a different clinical course: slowly progressing, with malaise, sore throat, low-grade fever, and cough developing over 3–5 days. The main organisms responsible for atypical pneumonia are* M. pneumoniae* in older children and* C. pneumoniae* in infants.* Legionella *species are rarely identified in children [8, 12, 13].

The etiologic definition is difficult for many reasons, such as low yield of blood cultures, difficulty in obtaining adequate sputum specimens from younger children, frequent specimen contaminations by upper airways bacterial flora and invasiveness of pulmonary biopsy, lung aspiration, and bronchoalveolar lavage which are rarely performed [13]. However, over the last 10 years, there have been improvements in PCR techniques for viral identification on nasopharyngeal aspirates or secretion, and molecular assays are now commonly used in Europe and in the US.

Vaccines are the most effective strategy for prevention of pediatric CAP.* Haemophilus influenzae* type B (HiB) conjugate vaccine and 7-valent pneumococcal conjugate vaccines (PCV7) dramatically decreased the incidence of bacterial CAP after introduction of universal vaccination campaigns [14, 15]. PCVs have been included for some years in the immunization schedules of children in their first year of life in many countries and they have completely modified the burden of pneumococcal diseases among these children and their unvaccinated contacts of any age [16]. Currently, the polyvalent pneumococcal vaccine (PCV13) confers immunity to approximately 85% of serotypes responsible for most invasive pneumococcal diseases [17].

### 3.2. Same Pathogens, Same Treatment: International CAP Recommendations

Since its introduction during the 20th century, antibiotic therapy, along with vaccines, has decreased CAP mortality of 97% in developed countries [14]. Most of the time the choice of an antimicrobial agent is empirical and based on the most common etiologies for each age group, on the local prevalence of causative organisms, and on the presence of risk factors for atypical or resistant bacteria [18].

**Figure 1:**
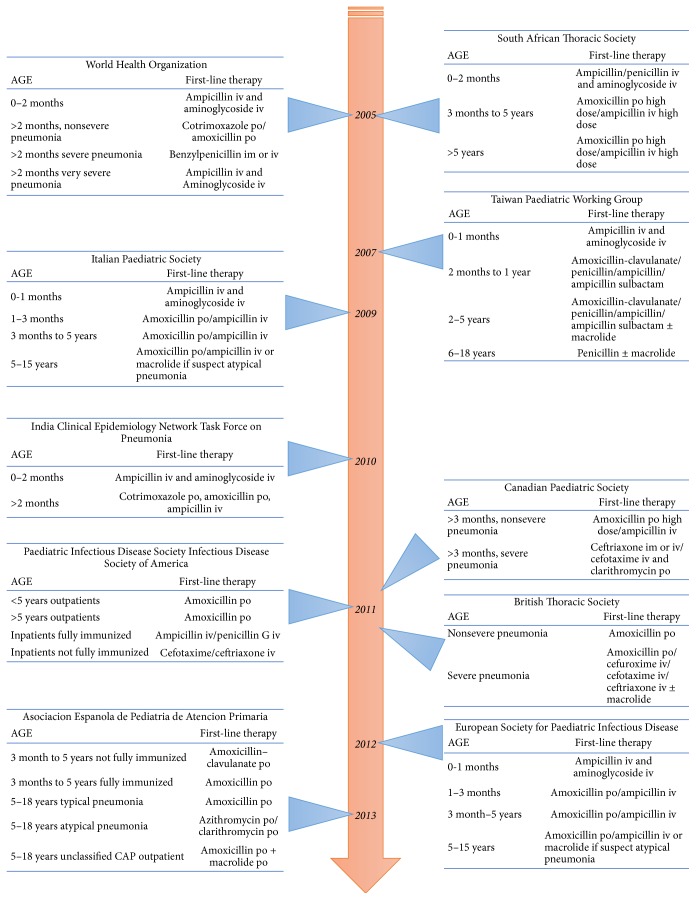
Pediatric CAP guidelines timeline [adapted by Berti et al., 2013 [19]].

During the last 10 years, many guidelines have defined the best antimicrobial regimen for CAP in children considering spectrum of activity, antimicrobial susceptibility, tolerability, bioavailability, safety, and cost [19, 20]. As already highlighted by other authors, these guidelines present some differences in treatment strategies, but almost all agree on the first-line therapy to administer in case of CAP (Figure 1) [19].

For infants < 2 months of age, the association with ampicillin and aminoglycosides is the most suggested therapy, ensuring coverage for Group B streptococci and Gram-negatives. In case of atypical pneumonia, in this period of life, because of the possibility of* Chlamydia trachomatis* infection, macrolides are recommended [3, 19, 21–23].

For all children > 3 months of age, the narrowest regimen with* S. pneumoniae* activity is suggested worldwide. Penicillin is the ideal first-line therapy, being a narrow-spectrum agent achieving therapeutic concentrations for* S. pneumoniae* in the lung up to MIC of 4 mg/ml [24]. However, due to its limited bioavailability, oral amoxicillin is reported as an equivalent and more feasible option [24, 25].

Despite general agreement on the agent, differences in dose and posology have been reported, varying according to pneumococcal resistance [19]. Indeed, beta-lactam effectiveness is time dependent and* S. pneumoniae *does not develop resistance through *β*-lactamase enzyme production, but through the alteration of the cell wall's antimicrobial targets (penicillin-binding proteins) [26]. Thus, in the setting of resistant* S. pneumoniae *serotype, higher concentration at the infection site is needed in order to saturate penicillin-binding proteins and to overcome resistance [27].

A study of children with pulmonary pneumococcal infection [28] provided data to develop a model for describing amoxicillin pharmacokinetics administered with different patterns: 50 mg/kg/day in two or three administrations daily. The resulting curve, integrated with* S. pneumoniae* MIC for amoxicillin, showed that, for intermediate resistant* S. pneumoniae* (MIC 4 mg/ml) CAP, the amoxicillin plasma concentration remained above the pneumococcal MIC level for about 4 hours. Therefore, amoxicillin administered every 8 hours maintains blood and lung concentrations that are above* S. pneumoniae* MIC for enough time to allow* S. pneumoniae* eradication. A longer interval between administrations (every 12 hours), in case of intermediate resistant serotypes, would not permit having a sufficient antimicrobial plasma concentration [28]. Similarly, penicillin G needs more frequent administrations than other beta-lactams, because of its shorter half-life [13].

Beta-lactam dose is the other key factor for pathogen eradication. Through the different guidelines, amoxicillin daily dose varies from 40–50 mg/kg to 90–100 mg/kg, with higher dosage recommended in areas with higher risk for antibiotic-resistant serotype, as in the US [13, 19]. In the same way, for inpatient parenteral therapy, higher doses of penicillin G or ampicillin are recommended [13].

The only two guidelines which suggest an aminopenicillin plus beta-lactamase inhibitor as first line are the Taiwan Pediatric Working Group and Asociacion Espanola de Pediatria de Atencion Primaria [29, 30]. Unlike the first one, in which aminopenicillin plus beta-lactamase inhibitor (e.g., amoxicillin-clavulanate) is suggested as first-line therapy for all children treated as outpatient, the Spanish guidelines recommend coamoxiclav only for children who are not fully immunized with conjugate vaccines for type B* H. influenzae* and for* S. pneumoniae*. Indeed, this population is at increased risk to develop a CAP by aggressive* S. pneumoniae *serotypes and other less common organisms, as* H. influenza*. Unlike Pneumococcus, type B and nontypeable* H. influenzae* became resistant to penicillin through the production of *β*-lactamase. Therefore, treatment with the association of amoxicillin with a *β*-lactamase inhibitor ensures a broader coverage [30]. It should be noted that the addition of a *β*-lactamase inhibitor does not change the amoxicillin kinetic curve; as a consequence, in order to treat a pneumococcal infection with the association of amoxicillin with clavulanate, the therapy should be administered every 8 hours [26].

The WHO guidelines are the only one suggesting cotrimoxazole as alternative to amoxicillin in outpatient treatment. This recommendation derived from evidence of no difference in treatment failure rates between amoxicillin and cotrimoxazole [31–33]. Despite concerns about the increase of* S. pneumoniae and H. influenzae *resistant to cotrimoxazole, as demonstrated by some authors [34], the reason for this indication is mainly attributable to economic factors. Indeed, for children <10 kg, the cost of a five-day treatment with amoxicillin is higher than the same duration on cotrimoxazole [35–37].

No guidelines recommend oral cephalosporins as first-line therapy. Indeed, pharmacokinetic and pharmacodynamic studies showed that none of the available oral cephalosporins is able to exceed the pneumococcal MIC for more than 50% of the time between two administrations [26]. Moreover, recent US data on* S. pneumoniae* susceptibility to cefdinir and cefuroxime indicated only 70% to 80% efficacy, compared with 84% to 92% amoxicillin efficacy [38, 39].

The only cephalosporin that has been demonstrated superior to penicillin in* S. pneumoniae* eradication, even if resistant, is ceftriaxone [40]. No microbiologic failures have been reported for* S. pneumoniae* with ceftriaxone MIC of 4.0 mg/mL [13, 41]. Thus, ceftriaxone or cefotaxime in standard doses is suggested by all guidelines as alternatives in case of first-line treatment failure, severe clinical conditions, or not fully immunized children [3, 7, 13, 21–23, 29, 30, 41].

Due to high prevalence of macrolide resistance circulating strains of* S. pneumoniae*, macrolides are not recommended as empiric therapy for CAP. Their use is suggested only when atypical etiology is suspected or in case of persistence of symptoms despite beta-lactams administration [7, 13, 42]. This strict indication for macrolides use derives from the evidence that* Mycoplasma* lower respiratory tract infection (LRTI) has a high rate of spontaneous clinical remission and the use of azithromycin has been associated with the selection of resistant organisms because of its prolonged serum elimination half-life [13]. Moreover, no significant benefits of antibiotic treatment in* M. pneumonia* infection have been documented [37].

For complicated pneumonia (i.e., moderate parapneumonic effusion and necrotizing pneumonia), antimicrobial therapy must be broadened to cover less common but highly aggressive pathogens as* Streptococcus pyogenes* and* S. aureus*. As for* S. pneumoniae*, macrolides cannot be considered an effective empiric therapy because of the high level of resistance [13].

Despite the fact that no penicillin or cephalosporin resistance has been reported for* S. pyogenes*, some authors suggest that, in case of concomitant symptoms attributable to toxic shock syndrome, combination therapy with clindamycin decreases the severity of symptoms [43]. In fact, since clindamycin inhibits protein synthesis (by binding the 50S subunit of the bacterial ribosome), it inhibits the production of* S. aureus* toxins, resulting in a lower inflammatory reaction. Clindamycin may be bacteriostatic or bactericidal depending on the organism and drug concentration and is indicated by US guidelines as a good option for both methicillin susceptible* S. aureus* (MSSA) and community-acquired methicillin-resistant* S. aureus* (CA-MRSA) strains [13].

Nowadays almost all MSSA have penicillin resistance which can be overcome with the addition of a *β*-lactamase inhibitor or through penicillinase-resistant beta-lactams, such as oxacillin or first-generation cephalosporins. MRSA strains have mecA gene that encodes penicillin-binding protein 2a, an enzyme that has low affinity for beta-lactams, leading to resistance to all antibiotics active against MSSA. During the last decade, both community-associated and hospital-acquired infections with MRSA have increased. MRSA, accounting for 20%–40% of all hospital-acquired pneumonia (HAP) and ventilator-associated pneumonia (VAP), have demonstrated a rapid increase as cause of pneumonia even in patients without exposure to the healthcare system [44]. This CA-MRSA has become an important cause of CAP complicated by empyema and necrosis [45].

Since erythromycin resistance predicts inducible clindamycin resistance in many isolates, a* D*-test to assess clindamycin susceptibility should always be performed. In case of* D*-test positivity, the use of clindamycin should be avoided, since it is highly possible that the organism will become resistant during the infectious process, especially in high-inoculum infections such as empyema [45]. On the other hand, all CA-MRSA strains are susceptible to vancomycin, which is considered by all guidelines as the drug of choice if MRSA is suspected [7, 13]. Although linezolid has been recently demonstrated as efficient as vancomycin for the treatment of MRSA pneumonia, its use should be considered as a second-line treatment for cost consideration (linezolid costs >10 times more than vancomycin) and because linezolid-resistant MRSA has already been described [46, 47].

### 3.3. Different Countries, Same Treatment?

**Figure 2:**
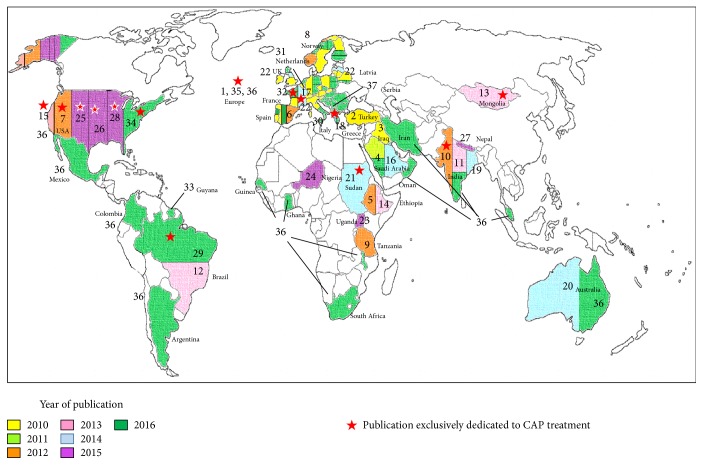
World map of papers on CAP treatment in children stratified by year of publication.

A worldwide review about CAP antimicrobial therapy in children includes 37 studies about antibiotics prescriptions in 50 countries published since 2010. The results are shown in Table 1 and Figure 2. Even if the studies were different in design and study population, their results give a good picture of the antibiotic prescription patterns in different environments, and they show the global heterogeneity in the application of the guidelines for the treatment of childhood pneumonia.

In fact, the first important result of our review is that the correct implementation of the guidelines is not confined to specific areas but may be variable even inside the same country. For example, Iroh Tam et al., through a 2-year retrospective study on hospitalized children with CAP in six US centres, showed that the most used antibiotics were third-generation cephalosporins (73%), and only 1% of the patients received amoxicillin. These findings during the first 2 years after US guidelines publication led the authors to recommend more strategies for educating healthcare providers [71]. On the other hand, Thomson et al. in another retrospective study set in an US hospital, with the same population (hospitalized children between 3 months old and 18 years old) in a 15-month period (May 2011–July 2012), had an opposite result, reporting that 63,6% of the pediatric CAP were treated with aminopenicillins and only 16.8% with third-generation cephalosporins [80].

We found a similar situation comparing studies from France [63, 78] and India [57, 65].

Interestingly, in France our data about CAP prescriptions derive from two different settings. Launay and colleagues investigated antimicrobial prescriptions and recommendations adherence in a French Emergency Pediatrics Department through a prospective two-period study, including all children aged one month to 15 years. The results were encouraging, with an increase of recommendation compliance from 18.8% to 48% between 2009 and 2012, and a consequent increase of amoxicillin monotherapy prescription from 54.2% to 71% [78]. Dubos et al., on the other hand, give us a picture of CAP antimicrobial prescriptions through general practitioners (GPs), private pediatricians, and pediatric fellows. The results of the standardized questionnaire submitted to every participant showed that CAP guidelines were insufficiently followed, with high rate of amoxicillin/clavulanate prescriptions (amoxicillin in monotherapy was prescribed in only 29% of cases, for 54% of cases associated with clavulanic acid) [63].

In India, in addition, we found some of the lowest rates of prescription on aminopenicillins as single therapy. Choudry and Bezbaruah, in a prospective observational study based in a university hospital in Assam, including inpatients up to 12 years, reported 0% use of penicillin as single therapy in cases of pediatric pneumonia. The therapy mostly used (54% of cases) was the combination of amoxicillin/clavulanate [58]. Another prospective study by Moinuddin et al. was conducted over 9 months in 2012, in two hospitals in Bangalore. The most widely used therapy was amoxicillin + clavulanate (43,8%), with third-generation cephalosporins as the most prescribed class (ceftriaxone 36.2%, cefotaxime 21%). Penicillin in single therapy accounted only for 1% of prescriptions [57].

Cephalosporins were often reported to be the class with higher rates of prescription for CAP treatment, as reported by many centres in different countries, like Ethiopia [60], Saudi Arabia [62], Nepal [73], Serbia [83], Sudan [67], US [54, 71, 74], Italy [76], and other European countries [48, 82].

Feleke and colleagues conducted their 5-month prospective study in a large government hospital in Ethiopia. The study includes all children admitted in that period and CAP accounted for 56.3% of all drug prescriptions. Ceftriaxone was the most prescribed drug (43.5%) followed by gentamicin (25.6%), and penicillin and ampicillin ranked the third and fourth place [62]. In a retrospective study by Zec et al., during a 6-month period in 2014, first- and third-generation cephalosporins were given to children with CAP in 40.4% and 31.7% of cases, respectively. Penicillin was used in 25% of cases [83]. In an Italian 1-day point-prevalence survey on antimicrobial use in hospitalized neonates and children in 2012, the main indication for treatment in children was LRTI (34%), with higher prevalence of third-generation cephalosporins (43.3%) followed by macrolides accounting for 26.8%. No ampicillin/amoxicillin prescription was reported [76].

Association of aminopenicillins was found to be often prescribed: amoxicillin + clavulanate was reported to be the most used therapy by studies conducted in Saudi Arabia [51], France [63], and India [58], and a study conducted in Iraq, by Younis, reported that ampicillin + cloxacillin, alone and in combination, accounted for 50% of the antibiotic prescriptions for the children with respiratory tract infections [50].

One study, in particular, reported a high rate of prescriptions of macrolides. It was conducted in Norway, by Fossum and colleagues, and included the prescriptions of general practitioners in case of respiratory tract infections in patients < 6 years. They found that macrolides were prescribed in 44% of the cases of pneumonia, more than penicillin V, which was used in 31%, and that extended spectrum penicillin accounted for 24% of the prescriptions [55].

Studies on the appropriateness of prescriptions or prescriber behavior were also found. In addition to the aforementioned French study, Maltezou et al. showed how Greek private-practice pediatricians guidelines compliance is only around 30.6% [64]. Moreover, Ceyhan et al., in a multicenter point-prevalence survey with respiratory infection as main diagnosis, showed how cephalosporins and penicillin (most of the time combined with b-lactamase inhibitors) were improperly prescribed in 36.1% and 43.7% of cases, respectively. These analyses highlighted how, even now, adherence to guidelines is still low. On the other hand, Usonis and colleagues through a questionnaire developed and distributed by the CAP Pediatric Research Initiative (CAP-PRI) working group and distributed across Europe showed high adherence to CAP guidelines, with a high prescription rate of narrow-spectrum penicillin for inpatients (amoxicillin (32%) and ampicillin (37%)) and outpatients (amoxicillin (84%)) [81].

An encouraging result is that almost a half (15/38) of the studies included in this review reported high rates of single therapy aminopenicillin or penicillin prescriptions. These studies were conducted in Brasil [75], Guyana [79], India [65], Mongolia [6], Nigeria [70], Tanzania [56], USA [80], Uganda [69], and France [78], showing that the current guidelines are applied in both developed and developing countries. The study by Awor et al. in Uganda in 2015 offers an important cause for reflection, since it shows that adherence to guidelines may be successfully implemented even in a nonhospital environment. In their 8-month quasi-experimental analysis, they investigated the visits and the prescriptions made by drug shop sellers, underlining how this class of health workers plays an important role in providing healthcare to populations in rural areas. Their result is that 91% of the children with pneumonia that were visited by drug shop sellers received amoxicillin, the highest rate of its prescription among all the studies included in this review [69].

Some data of antimicrobial prescriptions have been derived from point-prevalence surveys (PPS), including Australia [66, 82], Mexico, Colombia, Argentina, Singapore, and European countries [48, 49, 81, 82]. CAP was not the only analyzed disease, but the LRTI category was the most represented. Even though antimicrobial prescriptions were not specific only to CAP, PPS data were similar to the results of those other studies that were performed in the same country, but specifically designed for CAP.

Another interesting result is that the development of a local antimicrobial stewardship program could reduce inappropriate antimicrobial use and bacterial resistance, enhance patients' safety, and lower drug costs [84]. Moreover, global PPS could be a reliable and feasible tool for monitoring antimicrobial prescriptions all over the world.

Finally, it is also worthy of notice how data from certain countries were not available despite interest in the improvement of antibiotic prescription. For example, we did not find any report about pediatric CAP antibiotic treatment in Canada, even extending the research to 2005–2010. Likewise, we did not find any study set in other important countries, like China and Russia. It is worth remembering that the reduction of antimicrobial therapy and of microbial resistance is a global issue, and global effort is required in order to improve antibiotic prescription and administration practice.

## 4. Conclusions

In the last 10 years, many guidelines on the optimal treatment for childhood CAP have been published, with the aim of optimizing pediatric CAP antibiotic prescriptions. Our review demonstrates that the implementation of these guidelines is still limited but also that achieving the optimal prescription is possible and can be done in both developed and developing countries.

## Figures and Tables

**Table 1:** Papers on CAP antibiotic treatment in children from 2010 to 2016.

	Authors year of publication [ref.]	Country	Study design	Treated infections (% of pneumonia)	Population: age in/outpatient	Most prescribed antibiotics (%)
(1)	Amadeo et al. (2010) [48]	Europe	Multicenter, 2-day PPS on abx prescriptions	Various (respiratory tract infection: 30%)	<18 y inpatients	Third-generation cephalosporins (18%)

(2)	Ceyhan et al. (2010) [49]	Turkey	Multicenter, cross-sectional, 1-day PPS	Various (29.4%)	<18 y inpatients	Cephalosporins (22.1%), penicillin (20.5%) 56% inappropriate prescriptions

(3)	Younis (2010) [50]	Iraq	6-month, multicenter, prospective, observational study	Various (20%)	6 m–16 y inpatients	Ampicloxacillin (50%)

(4)	Mohajer et al. (2011) [51]	Saudi Arabia	1-month, retrospective, cross-sectional study on pharmacy prescriptions	Various (16.2%)	<12 y inpatients	Cephalosporin <1 yr (44.6%), coamoxiclav 1–5 years (35.4%), and 5–12 years (35.8%)

(5)	Bergicho et al. (2012) [52]	Ethiopia	1-month, single-center observational retrospective study on abx prescriptions	Various (9.27%)	<18 y inpatients	Cotrimoxazole (18.87%) Amoxicillin (14.5%)

(6)	Borrás Novell et al. (2013) [53]	Spain	A 1-year, prospective multicenter study including patients seen in PED on day 14 of each month who required hospitalization with systemic abx	Various (29.4%)	<18 y inpatients	Cefotaxime (27.8%), coamoxiclav (23.4%)

(7)	Brogan et al. (2012) [54]	USA	5-year, multicenter, retrospective cohort study from the Pediatric Health Information System (PHIS)	100%	1–18 y inpatients	Cephalosporins (40.4%)

(8)	Fossum et al. (2013) [55]	Norway	1-year, observational study primary care records	All respiratory tract infection (2.4%)	<6 y outpatients	Macrolides (44%)

(9)	Gwimile et al. (2012) [56]	Tanzania	7-month, single-center, cross-sectional descriptive hospital based study	Various (41%)	1 m–5 y inpatients	Penicillin (47.9%)

(10)	Moinuddin et al. (2012) [57]	India	9-month, prospective treatment charts review	100%	<18 y inpatients	Third-generation cephalosporins (57.2%)

(11)	Choudry and Bezbaruah (2013) [58]	India	1-month, single-center observational prospective study on abx prescriptions	Various (17%)	<12 y inpatients	Coamoxiclav (35%) Ceftriaxone (29%)

(12)	De Sá Del Fiol et al. (2013) [59]	Brazil	12-month, cross-sectional study on questionnaire on abx prescriptions in two Primary Health Centres	Various (3.13%)	<9 y outpatients	Penicillin (73.13%)

(13)	Dorj et al. (2013) [6]	Mongolia	10-week observational prospective study on written abx prescriptions of community pharmacies in rural and urban areas	100%	Adults and children outpatients	Aminopenicillins (16%)

(14)	Feleke et al. (2013) [60]	Ethiopia	6-month, prospective, cross-sectional study on patients charts	Various (56.3%)	<10 y inpatients	Ceftriaxone (43.50%)

(15)	Neuman et al. (2013) [61]	USA	Data were obtained from the National Hospital Ambulatory Medical Care Survey (NHAMCS) for ED visits from 2001 through 2009 for children with CAP	100%	Adults and children outpatients	Cephalosporin (35%) Macrolides (36%)

(16)	Alakhali and Shaik_Mohammad (2014) [62]	Saudi Arabia	2-month, observational, retrospective study on abx prescriptions	Various (9.7%)	<12 y inpatients	Cephalosporin (52%)

(17)	Dubos et al. (2014) [63]	France	A phone survey with a standardized questionnaire submitted randomly to GPs, pediatricians, and pediatric fellows	100%	<18 y outpatients	Coamoxiclav 54% Amoxicillin 29%

(18)	Maltezou et al. (2014) [64]	Greece	A standardized questionnaire distributed to 520 private-practice pediatricians	100%	<18 y outpatients	Compliance with the first-line recommended antibiotic was 30.6% for CAP

(19)	Mishra et al. (2014) [65]	India	Single-center, prospective, interventional study	Various (LRTI: 17.9%)	1 m–16 y outpatient	Amoxicillin (44%)

(20)	Osowicki et al. (2015) [66]	Australia	Multicentre, single-day, hospital-wide PPS	Various (LRTI: 22%)	<18 y inpatients	Narrow-spectrum penicillin (18%) *β*-lactam–*β*-lactamase inhibitor combinations (15%)

(21)	Salih et al. (2014) [67]	Sudan	12-month, cross-sectional study on abx prescriptions	100% (severe)	2 m–5 y inpatients	Coamoxiclav (22.1%) Cephalosporins: (i) Ceftriaxone (20.2%) (ii) Cefuroxime (19.7%)

(22)	Sviestina et al. (2014) [68]	France, Latvia, and UK	Multicenter, 1-day PPS on abx prescriptions ##	Various: LRTI Latvia (26.2%), France (11.8%), UK (9.3%)	<18 y inpatients	UK: piperacillin/tazobactam (32%), coamoxiclav (26%) Latvia: amoxicillin (30%), ceftriaxone (21%) France: coamoxiclav (21%), amoxicillin (17%)

(23)	Awor et al. (2015) [69]	Uganda	All drug shops in the intervention area were included and all child visits in 8 months were analyzed	Various (45%)	<7 y outpatients	Amoxicillin (91%)

(24)	Fadare et al. (2015) [70]	Nigeria	7- month, cross-sectional study using medical records	Various (respiratory tract infections: 53.7%)	<5 y outpatients	Amoxicillin (52.4%) Coamoxiclav (19%)

(25)	Iroh Tam et al. (2015) [71]	USA	Multicenter, retrospective study (six hospitals) on medical records with pneumonia	100%	2 m–18 y inpatients	Third-generation cephalosporins (72%)

(26)	Milner et al. (2015) [72]	USA	2-year multicenter retrospective cohort study	100%	3 m–18 y	Emergency department providers prescribed narrow-spectrum therapy 27% of the time

(27)	Thapaliya et al. (2013) [73]	Nepal	6-month, single center, retrospective study on medical charts	Various (22.5%)	<13 y inpatients	Cephalosporins (ceftriaxone 49.3%, cefotaxime 26.2%)

(28)	Williams et al. (2015) [74]	USA	6-month multicenter, prospective, population-based, active surveillance of CAP hospitalizations among children pre: 1–9%, post: 15.2%	100%	3 m–18 y inpatients	Cephalosporins pre (52.8%)

(29)	Fonseca Lima et al. (2016) [75]	Brazil	3-year, single-center, cross-sectional study	100%	1 m–5 y inpatients	Ampicillin 62.17%

(30)	De Luca et al. (2016) [76]	Italy	1-day PPS on abx prescriptions ##	Various (LRTI: 22.1% of children, 2.3% of neonates)	<18 y inpatients	Cephalosporins (43.3%)

(31)	Ivanovska et al. (2016) [77]	Netherlands	3-year, retrospective, observational study, deriving data on diagnoses and prescriptions from the electronic health records-based NIVEL Primary Care Database	Respiratory tract infection (pneumonia 5.8–7.1%)	<18 y outpatients	Amoxicillin: 2010 (60.4%), 2011 (66.9%), and 2012 (63%)

(32)	Launay et al. (2016) [78]	France	Multicenter, prospective two-period study using data from the French pneumonia network	100%	1 m–15 y inpatients	First period: amoxicillin 58.1% Second period: amoxicillin 71.0%

(33)	Sharma et al. (2016) [79]	Guyana	1-year, retrospective chart review of pediatric patients seen in the emergency department	Various (RTI: 19.5%)	1 m–13 y outpatients	Amoxicillin 33.6%

(34)	Thomson et al. (2015) [80]	USA	15-month, single-center, retrospective cohort study	100%	3 m–18 y inpatients	Aminopenicillins (63.6%) Third-generation cephalosporins (16.8%)

(35)	Usonis et al. (2016) [81]	Europe	Snapshot prospective study based on a questionnaire developed and distributed by the CAP Paediatric Research Initiative (CAP-PRI) working group and distributed across Europe	100%	<18 y inpatients and outpatients	Inpatients: amoxicillin (32%), ampicillin (37%) Outpatients: amoxicillin (84%)

(36)	Vesporten et al. (2016) [82]	Africa, Asia, Oceania, Latina America, North America and Europe	1-day PPS on abx prescriptions ##	Various (LRTI 18.7%)	<18 y inpatients	Third-generation cephalosporins: Eastern Europe (37.5%) and Asia (28.6%), fourth-generation cephalosporins in North America (13.3%). Narrow-spectrum (b- lactamase sensitive penicillin 11% in Africa and 4.3% in Northern Europe)

(37)	Zec et al. (2016) [83]	Serbia	Single-center, 6-month, retrospective study on medical charts	100%	1 m–6 y inpatients	Cephalosporins (cefazolin 40.4%, third-generation cephalosporins 31.7%)

^##^Data from Antibiotic Resistance and Prescribing in European Children (ARPEC) project.

## Abbreviations

CAP: Community-acquired pneumonia
RSV: Respiratory syncytial virus
HiB: Type B* Haemophilus* influenzae
PCV, PCV7, and PCV 13: Pneumococcal conjugate vaccine
MIC: Minimal inhibitory concentration
LRTI: Lower respiratory tract infection
MSSA: Methicillin-sensitive* Staphylococcus aureus*
MRSA: Methicillin-resistant* Staphylococcus aureus*
CA-MRSA: Community-acquired methicillin-resistant* Staphylococcus aureus*
HAP: Hospital-acquired pneumonia
VAP: Ventilator-associated pneumonia.

## Additional Points

*Availability of Data.* The datasets used and/or analyzed during the current study are available from the corresponding author on reasonable request.
